# Supplementary material for: A diffusion anisotropy descriptor links morphology effects of H-ZSM-5 zeolites to their catalytic cracking performance
Source: Commun Chem. 2021 Jul 16;4:107. doi: 10.1038/s42004-021-00543-w (PMC9814363; doi:10.1038/s42004-021-00543-w)
Supplement: Supplementary file 1 — Supplementary Information [file 42004_2021_543_MOESM1_ESM.pdf]

Supplementary Information

A diffusion anisotropy descriptor links morphology effects of H-ZSM-5 zeolites to their catalytic cracking performance

Liu *et al.*

Supplementary figures and tables

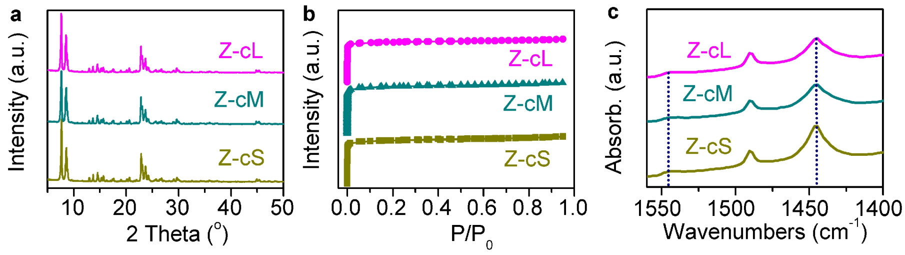

Supplementary Figure 1. Structures, textures, and acidity of the as-synthesized H-ZSM-5 samples. **a** *X*-ray diffraction patterns. **b** Ar physisorption isotherms. **c** Pyridine-adsorption IR spectra.

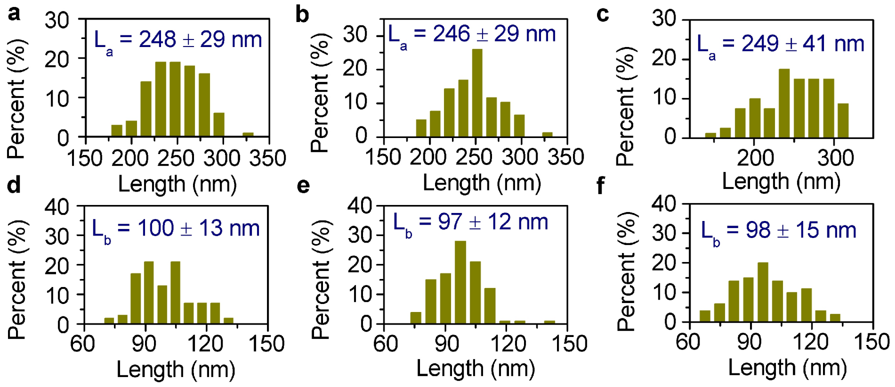

Supplementary Figure 2. Corresponding length distribution along *a*- and *b*- axis of H-ZSM-5 crystals. **a, d** Zc-S; **b, e** Z-cM; **c, f** Z-cL. The lengths of *a*- and *b*- axis over corresponding zeolites were counted by statistics of 100 specimens from the SEM images of Fig. 2a.

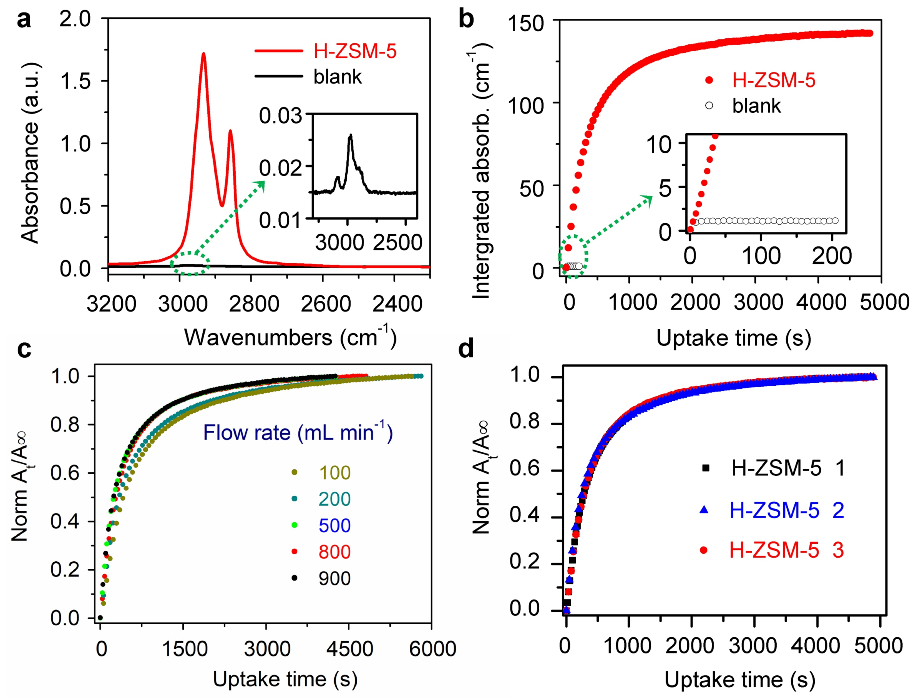

Supplementary Figure 3. Validation of the time-resolved *in situ* FTIR spectroscopy. The blank experimental FT-IR spectroscopy were conducted at a 0.1 vol%butene / 99.9 vol%N_2 mixture gas flow rate of 800 mL min^{-1} and a temperature of 373 K without H-ZSM-5 wafer in the IR flow cell. **a** The spectra of the typical infrared bands for butene molecule at the time of adsorption equilibrium: red line for H-ZSM-5 spectrum and black line for blank spectrum. **b** The variation in the integrated intensity of the typical infrared bands of the butene molecule through the fast-scanning FT-IR spectroscopy: red solid scatter for H-ZSM-5 wafer and black hollow scatter for blank experiment. It could be seen that the blank effect of IR setup can be eliminated at our measurement conditions. **c** The normalized uptake curves of H-ZSM-5 samples for butene molecule at a flow rates range of 100-900 mL min^{-1}. This fast flow rate, ensured that, firstly, no depletion of the sorbate in the intercrystalline voids occurred and, secondly, the heat of adsorption was rapidly dissipated, as was confirmed by appropriate measurements[1-3]. Depletion of the sorbate in the intercrystalline space and a non-isothermal situation as a consequence of evolution of the adsorption heat are, according to findings of Doelle and Riekert[4], main pitfalls in sorption kinetics measurements. **d** Repeatability of the time-resolved *in situ* FTIR spectroscopy. Our measurements were repeated at least third times to ensure repeatability of the data obtained, the experiments had a better repeatability.

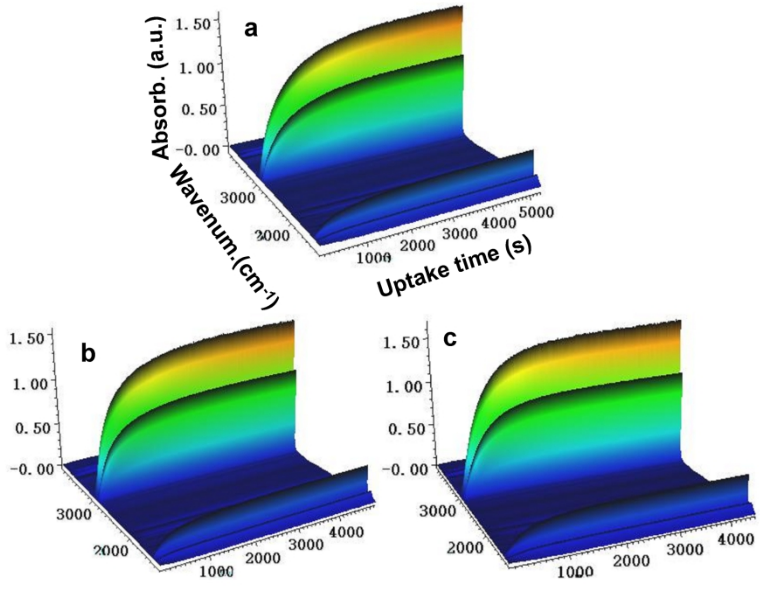

Supplementary Figure 4. The original uptake curves. **a** Z-cS, **b** Z-cM and **c** Z-cL. The normalized areas of the IR bands of butene and propene molecules at 2750 - 3150 cm^{-1} were used to quantify the relative concentrations of adsorbate in the zeolites.

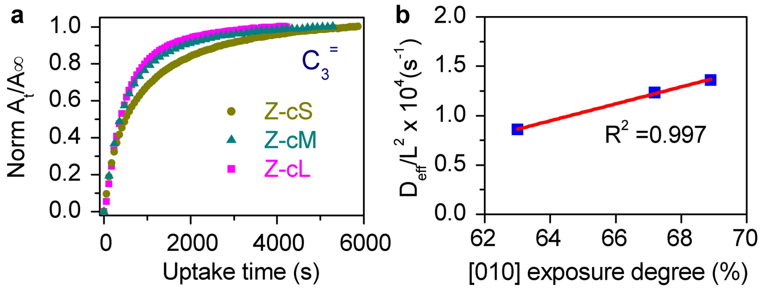

Supplementary Figure 5. Diffusion behaviors of $C_3^=$ in H-ZSM-5 zeolites. a Normalized uptake curves of $C_3^=$ over ZSM-5 samples with different length of *c*- axis. **b** Correlation of the [010] exposure degrees of ZSM-5 zeolites with the diffusion rates fitted by Eq.1. The diffusion rates increase almost linearly with the exposure degrees of [010] plane over H-ZSM-5 samples. The error bars are smaller than the data points.

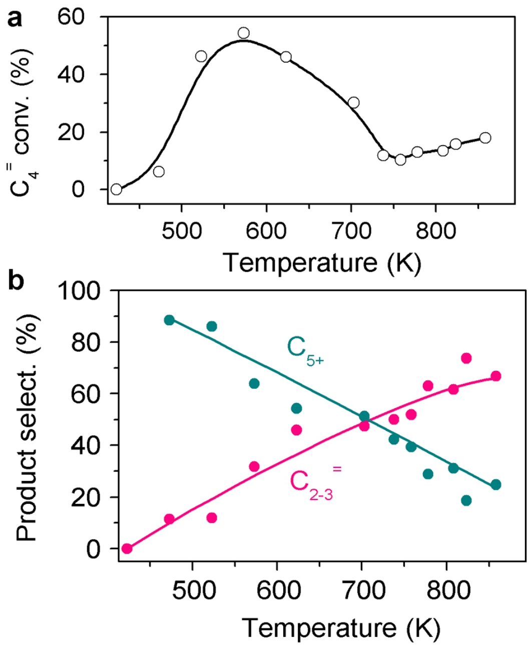

Supplementary Figure 6. Catalytic behaviours of H-ZSM-5 catalysts for the conversion of $C_4^=$ at different temperatures. a $C_4^=$ conversions. **b** Products selectivity. Reaction conditions: *M* (catalyst) = 0.1 g; *P* = 1.6 Bar; *T* = 423 - 858 K; C_4H_8/N_2 = 1 vol%/99 vol%, *F* = 100 mL min^{-1}; Time on stream, 4 h.

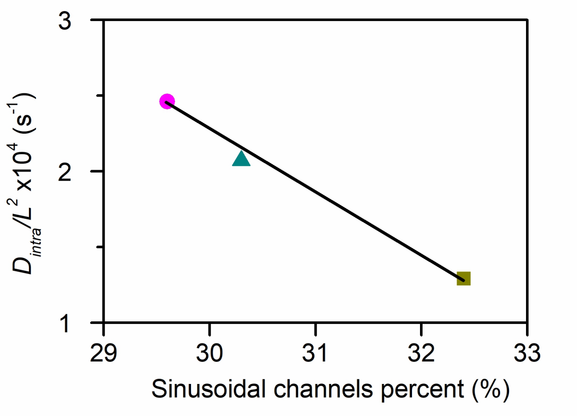

Supplementary Figure 7. Correlation of sinusoidal channels percents computed in table 1 with the intracrystalline diffusion rates (D_{intra}/L^2) of butene molecule. The error bars are smaller than the data points.

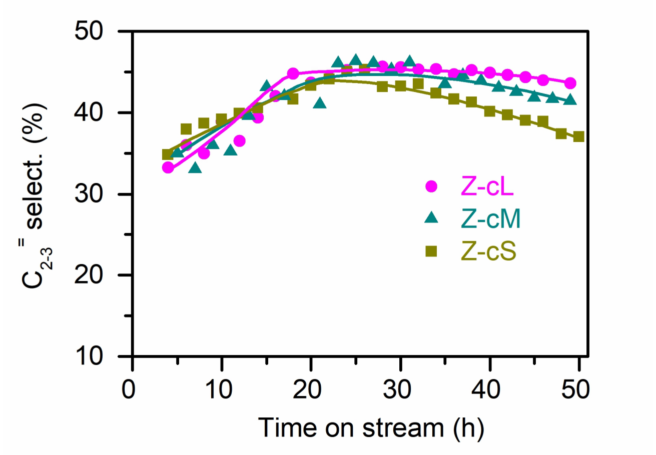

Supplementary Figure 8. The $C_{2\text{-}3}^{=}$ selectivity for C_4 olefins catalytic cracking reactions over Z-cS, Z-cM and Z-cL catalysts. Reaction conditions: M (catalyst) = 0.3 g; P = 1.6 Bar; T = 823 K; F = 15 mL h^{-1}; Time on stream, 50 h.

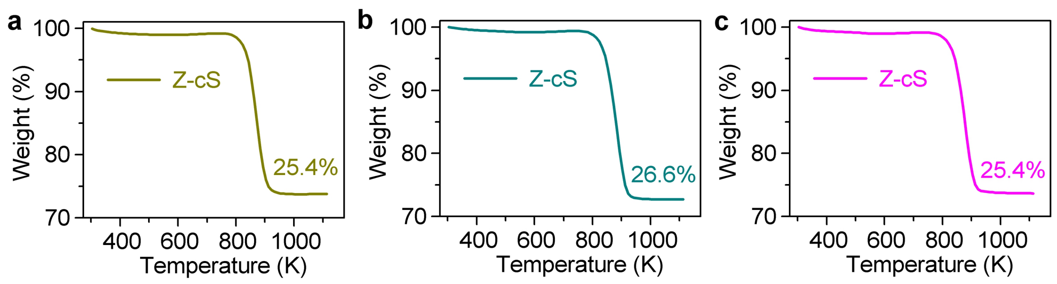

Supplementary Figure 9. The TGA of the three ZSM-5 catalysts after reaction for 50 h. a Z-cS; **b** Z-cM; **c** Z-cL.

Supplementary Table 1. Detailed textural parameters and Brønsted acid sites of the as-synthesized H-ZSM-5 samples.

Samples	S_{BET} [a] $m^2\ g^{-1}$	S_{micro} [b] $cm^2\ g^{-1}$	$S_{external}$ [c] $cm^2\ g^{-1}$	S_{meso} [d] $cm^3\ g^{-1}$	V_{total} [e] $cm^3\ g^{-1}$	V_{micro} [f] $cm^3\ g^{-1}$	V_{meso} [g] $cm^3\ g^{-1}$	Si/Al ratio[h]	Brønsted acid sites[i]
Z-cS	365	337	28	29	0.169	0.143	0.027	288	0.323
Z-cM	393	365	29	29	0.183	0.160	0.027	301	0.283
Z-cL	339	316	22	23	0.154	0.134	0.021	297	0.314

[a] BET surface area.

[b] Micropore area evaluated by the t-plot method.

[c] t-plot external surface area.

[d] BJH desorption method.

[e] Single point adsorption total pore volume of pores, P/P_0=0.994.

[f] Pore volume for micropores evaluated by the t-plot method.

[g] Pore volume for mesopores evaluated by BJH desorption.

[h] Determined by ICP analysis.

[i] Determined by Pyridine-adsorption IR spectra.

Supplementary Table 2. The unit cell parameters and the degree of exposed pore channels per unit cell area in corresponding crystal plane.

Crystal-facets[a]	Unit cell D_1 (Å)	Unit cell D_2 (Å)	Unit cell area (Å^2)[b]	Pore amounts unit cell^{-1}	Pore amounts[c] nm^{-2}
[010]	20.07	13.42	269.34	2	0.743
[100]	19.92	13.42	267.33	2	0.748
[101]	19.92	24.14	480.87	2	0.416

[a] The lattice parameters for the crystal unit cell have been decided from *X*-ray diffraction methodology, as shown in IZA database, and the diameters of *a-, b-, c-* directions in ZSM-5 unit cell are considered to 20.07, 19.92, and 13.42 Å in the *Pnma* space group (orthorhombic).

[b] S(one unit cell area) = Unit cell D_1 * Unit cell D_2.

[c] Pore amounts per nm^2 = Pore amounts per unit cell / S(one unit cell area). The n[010], n[100] and n[101] are used for representing the pore amounts per nm^2 of corresponding crystal facet.

Supplementary Table 3. The apparent diffusion rates over Z-cS and Z-cL at different temperature.

Temperature	D_{eff}/L^2 (s^{-1})	
	Z-cS	Z-cL
323 K	0.35E-4	0.57E-4
373 K	1.07E-4	1.75E-4
398 K	1.76E-4	2.44E-4
423 K	3.06E-4	3.36E-4

Supplementary Table 4. The diffusion coefficients for $C_3^=$ molecule along x and y directions (*$D_{self\text{-}xx}$* and *$D_{self\text{-}yy}$*) derived from the slope of corresponding curves of the molecular dynamics (MD) simulations at different temperature.

T K	$D_{self\text{-}xx1}$	$D_{self\text{-}yy1}$	$D_{self\text{-}xx2}$	$D_{self\text{-}yy2}$	$D_{self\text{-}xx3}$	$D_{self\text{-}yy3}$	Average $D_{self\text{-}xx}$	Average $D_{self\text{-}yy}$	$D_{self\text{-}xx}/D_{self\text{-}yy}$
323	36.73	97.08	37.27	93.52	27.86	93.49	33.95	94.70	0.358
373	27.95	112.10	41.57	70.46	53.86	64.77	41.13	82.44	0.557
423	64.09	95.23	60.71	129.16	50.71	80.51	58.50	101.63	0.591

All of the simulations were parallelly calculated for three time, the average *MSD* (mean square displacement), $D_{self\text{-}xx}$ and $D_{self\text{-}yy}$ was chosen in this work.

Supplementary Table 5. The diffusion coefficients for $C_4^=$ molecule along x and y directions (*$D_{self\text{-}xx}$* and *$D_{self\text{-}yy}$*) derived from the slope of corresponding curves of the molecular dynamics (MD) simulations at different temperature.

T K	$D_{self\text{-}xx1}$	$D_{self\text{-}yy1}$	$D_{self\text{-}xx2}$	$D_{self\text{-}yy2}$	$D_{self\text{-}xx3}$	$D_{self\text{-}yy3}$	Average $D_{self\text{-}xx}$	Average $D_{self\text{-}yy}$	$D_{self\text{-}xx}/D_{self\text{-}yy}$
323	16.65	64.07	30.00	86.90	31.36	95.09	26.00	82.02	0.312
373	35.94	96.46	37.96	72.21	50.48	128.15	41.46	98.94	0.431
423	33.25	74.52	52.25	88.69	63.32	125.49	49.61	96.23	0.513

All of the simulations were parallelly calculated for three time, the average *MSD* (mean square displacement), $D_{self\text{-}xx}$ and $D_{self\text{-}yy}$ was chosen in this work.

Supplementary Table 6. Detailed textural parameters of the H-ZSM-5 samples after 50 hours of reaction.

Samples	S_{BET} [a] $m^2\ g^{-1}$	S_{micro} [b] $cm^2\ g^{-1}$	$S_{external}$ [c] $cm^2\ g^{-1}$	S_{meso} [d] $cm^3\ g^{-1}$	V_{total} [e] $cm^3\ g^{-1}$	V_{micro} [f] $cm^3\ g^{-1}$	V_{meso} [g] $cm^3\ g^{-1}$
Z-cS	118	101	16	14	0.065	0.042	0.022
Z-cM	178	160	18	16	0.092	0.067	0.025
Z-cL	182	165	17	16	0.094	0.069	0.024

[a] BET surface area.

[b] Micropore area evaluated by the t-plot method.

[c] t-plot external surface area.

[d] BJH desorption method.

[e] Single point adsorption total pore volume of pores, P/P_0=0.994.

[f] Pore volume for micropores evaluated by the t-plot method.

[g] Pore volume for mesopores evaluated by BJH desorption.

Supplementary References

1. Karge, H. G. & Niessen, W. A new method for the study of diffusion and counter-diffusion in zeolites. *Catal. Today* **8**, 451-485 (1991).
2. Karge, H. G. Infrared spectroscopic investigation of diffusion, co-diffusion and counter-diffusion of hydrocarbon molecules in zeolites. *C. R. Chimie* **8**, 303-319 (2005).
3. W. Niessen, PhD Thesis, Untersuchungen zur Diffusion und Gegendiffusion in Zeolithen mit Hilfe der FTIRSpektroskopie (Investigation of Diffusion and CounterDiffusion in Zeolites), University of Technology, Berlin, **1991**.
4. H.J. Doelle, L. Riekert, J.R. Katzer, Molecular SievesII, Proc. 4th Int. Conf. on Zeolites, Chicago, USA, April 18-22, 1977, ACS Symp. Ser. 40, Am. Chem. Soc., Washington, DC, **1977**, p. 401.
